# Exploring Plasma Coenzyme Q_10_ Status in Paediatric Dyslipidaemia

**DOI:** 10.3390/antiox13080966

**Published:** 2024-08-09

**Authors:** Beatriz Minguez, Mariela de Los Santos, Camila Garcia-Volpe, Cristina Molera, Abraham J. Paredes-Fuentes, Clara Oliva, Angela Arias, Helena Rodriguez-Gonzalez, Delia Yubero, Mireia Tondo, Carlos Santos-Ocaña, Silvia Meavilla, Rafael Artuch

**Affiliations:** 1 Gastroenterology and Nutrition Department, Hospital Sant Joan de Déu, 08950 Barcelona, Spain; beatriz.minguez@sjd.es (B.M.); marielamercedes.santos@sjd.es (M.d.L.S.); camila.garcia@sjd.es (C.G.-V.); cristina.molera@sjd.es (C.M.); silviamaria.meavilla@sjd.es (S.M.); 2 Division of Inborn Errors of Metabolism-IBC, Biochemistry and Molecular Genetics Department, Hospital Clínic de Barcelona, 08036 Barcelona, Spain; paredes@clinic.cat (A.J.P.-F.); cloliva@clinic.cat (C.O.); 3 Clinical Biochemistry Department, Institut de Recerca Sant Joan de Déu, 08950 Barcelona, Spain; angelayasmina.arias@sjd.es (A.A.); helena.rodriguez@sjd.es (H.R.-G.); 4 Genetic Department, Hospital Sant Joan de Déu, 08950 Barcelona, Spain; delia.yubero@sjd.es; 5 Department of Biochemistry, Hospital de la Santa Creu i Sant Pau, Biomedical Research Institute (IIB) Sant Pau, 08041 Barcelona, Spain; mtondo@santpau.cat; 6 Centre of Biomedical Investigation Network for Diabetes and Metabolic Diseases (CIBERDEM), 28029 Madrid, Spain; 7 Departamento de Fisiología, Anatomía y Biología Celular, Centro Andaluz de Biología del Desarrollo, Universidad Pablo de Olavide, 41013 Sevilla, Spain; csanoca@upo.es; 8 U703-U729 CIBERER, Instituto de Salud Carlos III, 28029 Madrid, Spain

**Keywords:** cholesterol, coenzyme Q10, hypercholesterolemia, hypobetalipoproteinemia, paediatric patients, dyslipidaemia

## Abstract

Coenzyme Q10 (CoQ) is a ubiquitous lipid with different biological functions. In blood, there is a close relationship between CoQ status and cholesterol, which strongly supports the study of both molecules simultaneously. The objective of this study was to evaluate plasma CoQ, lipoprotein concentrations and CoQ/Chol ratio in a cohort of paediatric patients with different types of dyslipidaemias. A total of 60 paediatric patients were recruited (age range: 7 months–18 years), including 52 with different types of hypercholesterolemia, 2 with isolated hypertriglyceridemia and 6 with hypobetalipoproteinemia. Plasma CoQ was analysed by HPLC with electrochemical detection, and lipoprotein and cholesterol concentrations by standard automated methods. The lowest CoQ values were detected in patients with hypobetalipoproteinemia and in two cases of liver cirrhosis. Mean CoQ values were significantly higher in hypercholesterolemic patients compared to controls (average values 1.07 µmol/L and 0.63 µmol/L) while the CoQ/cholesterol ratio did not show differences (170 vs. 163, respectively). Mean CoQ values were significantly lower in the group of patients with hypobetalipoproteinemia compared to controls (mean CoQ values of 0.22 µmol/L vs. 0.63 µmol/L, respectively), while those of CoQ/cholesterol did not show differences. Pearson’s correlation test showed a positive correlation between the CoQ and cholesterol values (r = 0.565, *p* < 0.001) and between the CoQ and the LDL cholesterol values (r = 0.610, *p* < 0.001). Our results suggest that it is advisable to analyse plasma CoQ and cholesterol concentrations in patients with hypobetalipoproteinemia and hypercholesterolemia associated with liver damage.

## 1. Introduction

**Figure 1:**
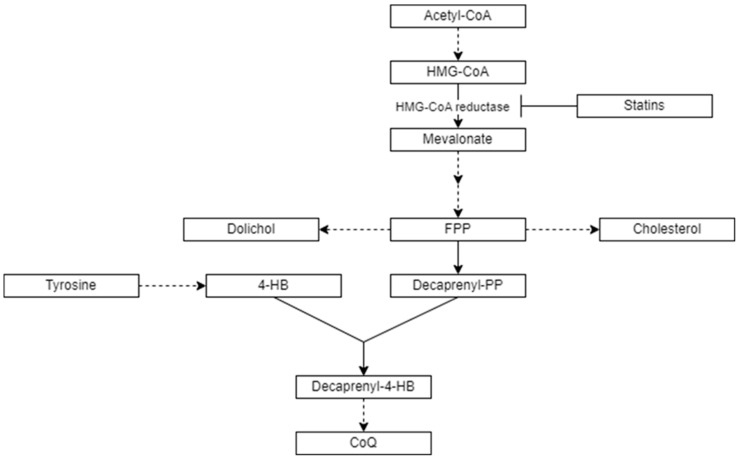
Main metabolic pathways related to CoQ and Chol biosynthesis. 4-hydroxybenzoate (4-HB), acetyl-coenzyme A (acetyl-CoA), farnesyl-pyrophosphate (FPP), decaprenyl-pyrophosphate (decaprenyl-PP), decaprenyl-4-hydroxybenzoate (decaprenyl-4-HB), and 3-hydroxy-3-methylglutaryl coenzyme A (HMG-CoA). Dashed arrows indicate that more than one enzymatic step is involved in the metabolism of the molecules.

Coenzyme Q10 (CoQ) is a ubiquitous lipid with different biological functions, such as acting in the mitochondrial respiratory chain as an essential electron transporter for ATP synthesis and serving as a lipophilic antioxidant, among others [1]. The benzoquinone ring of CoQ, which exerts antioxidant properties, is derived from tyrosine, and the polyprenyl side chain comes from acetyl-CoA through the mevalonate pathway (Figure 1), which is common to the synthesis of cholesterol (Chol) and other lipids [2]. 

In blood, there is a close relationship between CoQ status and Chol [3]. In fact, the CoQ supplementation effect on very-low-density lipoproteins (VLDLs), low-density lipoproteins (LDLs), and high-density lipoproteins (HDLs) has been characterised [4]. Furthermore, due to its antioxidant properties, CoQ has been shown to be very efficient in preventing LDL oxidation compared to other lipophilic antioxidants in the blood [5]. The blood CoQ status depends on liver biosynthesis and is also the result of dietary sources that can contribute up to 20% of the total amount [6]. Almost all tissues and cells can synthesise CoQ, and the degree of uptake of CoQ between blood and tissues remains largely unknown [7].

Taken together, identifying diseases characterised by chronic low plasma CoQ values may be important, as, for example, CoQ supplementation has been shown to be beneficial in improving the outcome of patients with chronic heart failure [8,9]. Some reports have delineated diseases associated with impaired CoQ status at the paediatric age, detected in some selected conditions, such as phenylketonuria, mevalonic aciduria, lysosomal storage diseases, Smith–Lemli–Opitz syndrome, and other conditions such as hyperthyroidism and cystic fibrosis [10,11,12,13].

The close relationship between cholesterol and CoQ strongly suggests that both molecules must be studied simultaneously. There are numerous studies investigating the potential effects of statins on cholesterol and CoQ, and the recommendation that arises is that CoQ supplementation seems advisable together with statin therapy [14,15,16]. Very few reports have been published on CoQ levels in paediatric dyslipidaemia [16].

With this background, the aim of this study was to evaluate plasma CoQ, lipoprotein concentrations and CoQ/Chol ratio in a cohort of paediatric patients with different types of dyslipidaemias, mainly hypobetalipoproteinemia, hypercholesterolemia, and hypertriglyceridemia. 

## 2. Materials and Methods

### 2.1. Subjects

A total of 60 paediatric patients were recruited (age range: 7 months–18 years, average = 9.6; SD = 4.2). Depending on the type of dyslipidaemia, patients were grouped according to their lipid profile in hypercholesterolemia (defined as Chol greater than 5.2 mmol/L; n = 52), isolated hypertriglyceridemia (defined as triglycerides greater than 2.3 mmol/L; n = 2), and hypobetalipoproteinemia (defined as apolipoprotein B less than 0.41 g/L and Chol < 2.2 mmol/L; n = 6). The etiological diagnosis of each patient when available, together with the biochemical data, is reported in Supplementary Table S1. Plasma CoQ and CoQ/Chol ratio results were compared with reference intervals established in a healthy control population (n = 42; age range: 2–18 years old, average = 11.1; SD = 5.5). For the other routine parameters (Table 1), we stated the reference intervals currently used in clinical laboratories. As the main exclusion criteria, patients with treatment of dyslipidaemia, such as statin therapy, tocopherol or other liposoluble vitamins, or CoQ supplementation, were not recruited. Plasma samples: Ethylenediamine tetraacetic acid (EDTA) and heparin blood samples were drawn to separate plasma by centrifugation (1500× *g*, 10 min, 4 °C). The samples were stored at −80 °C until CoQ analysis.

### 2.2. Samples and Laboratory Analysis

Blood samples were taken and processed following standard procedures. After centrifugation at 1500× *g* (10 min), serum was separated and used to quantify total Chol, triglycerides, HDL-Chol, and apolipoproteins A and B when indicated. These biomarkers were analysed using automated spectrophotometric and chemoimmunoluminescence methods. LDL cholesterol and VLDL cholesterol were calculated using the Friedewald formula and data were not considered in the 2 cases with hypertriglyceridemia and patients with mutations in the *APOC2* gene. A subsequent aliquot was stored at −80 °C for the quantification of CoQ by HPLC with electrochemical detection, as previously reported [17].

Genetic analysis was conducted by next-generation sequencing, using a clinical exome approach consistent with the simultaneous sequencing of the coding exons (exons, and 25 pb flanking intronic regions) over 5227 genes (Custom Comprehensive panel 17 Mb, Agilent Technologies, Santa Clara, CA, USA) or 6713 genes (TruSight One Sequencing Panel, Illumina, San Diego, CA, USA) with known clinical phenotype association according to the databases of Human Gene Mutation Database (HGMD), GeneTest.org and Online Mendelian Inheritance in Man (OMIM). Sequencing was performed in a NextSeq500 instrument (Illumina) and the data obtained were processed by a customised protocol adapted by Hospital Sant Joan de Déu Bioinformatics Unit, using the reference genome hg19. Candidate genetic variants were classified according to ACMG guidelines [18].

### 2.3. Statistical Analysis

All data are presented as mean ± SD (range). The Pearson correlation test was applied to search for associations among the different variables, Student’s *t*-test was used to compare data from hypercholesterolemic patients with those of controls, and multiple linear regression analysis was applied to explore the effect of different variables on CoQ values as the dependent variable. *p* < 0.05 is considered statistically significant. Statistical analysis was performed using software R version 4.3.0 software [19].

## 3. Results

**Figure 2:**
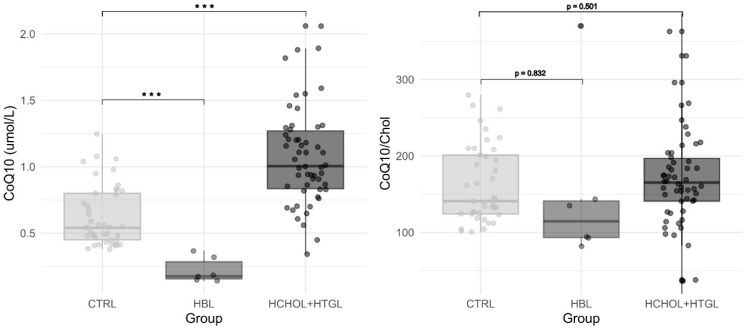
Box-plot representation of CoQ (**left graph**) and CoQ/Chol (**right graph**) results in patients with hypobetalipoproteinemia (HBL), hypercholesterolemia or hypertriglyceridemia (HCHOL + HTGL), and controls (CTRL). *p* values from Student’s *t*-test between groups are depicted (*** refers to *p* < 0.05).

Table 1 and Figure 2 show the biochemical characteristics of patients with different types of dyslipidaemias, compared to our reference values. 

In total, 7 out of the 60 patients in the series showed low total CoQ values (6 belonging to the hypobetalipoproteinemia group and 1 to the hypercholesterolemic group (Alagille syndrome). A total of 8 out of 60 showed a low CoQ/Chol ratio, corresponding to 3 patients with hypobetalipoproteinemia (heterozygous mutation in the *APOB* gene, hypofibrinogenemia caused by heterozygous mutation in the *FGG* gene, and chylomicron retention disease caused by homozygous mutation in the *SAR1B* gene) and 5 patients with hypercholesterolemia (1 patient with Alagille syndrome, 1 with liver cirrhosis, 1 with heterozygous *LDLR* mutations, and 2 of unknown aetiology). 

Mean CoQ values were significantly higher in hypercholesterolemic patients compared to controls (average values 1.07 μmol/L and 0.63 μmol/L, respectively; Student’s *t*-test *p* < 0.001) while the CoQ/Chol ratio did not show differences (167 vs. 163, respectively; *p* = 0.698). Mean CoQ values were significantly lower in the group of patients with hypobetalipoproteinemia compared to controls (mean CoQ values of 0.22 μmol/L vs. 0.63 μmol/L, respectively; Student’s *t*-test *p*< 0.001), while CoQ/Chol did not show differences (153 vs. 163; *p* = 0.832).

In the whole cohort of patients with dyslipidaemia, the Pearson correlation test showed a positive correlation between CoQ and Chol values (r = 0.565, *p* < 0.001) and between CoQ and LDL cholesterol values (r = 0.610, *p* < 0.001), but no significant correlation was observed with the patient’s age, triglycerides, or HDL and VLDL cholesterol subfractions. In the hypercholesterolemic group, only a positive correlation was observed between CoQ and LDL-Chol values (r = 0.32, *p* = 0.024). In the whole cohort of dyslipidemic patients, multiple linear regression analysis, with CoQ as the dependent variable, showed that LDL was the only variable contributing to the variability of CoQ variability (β = 0.169, *p* < 0.001, R2 = 0.315), while no significant effect was observed for triglycerides, age, and other subfractions of lipoproteins. The same analysis in the hypercholesterolemic group revealed that none of these variables had a statistically significant effect on CoQ values. Unlike the entire dyslipidemic cohort, LDL cholesterol did not emerge as a significant predictor of CoQ variability (β = 0.0868, *p* = 0.079, R2 = 0.045), although it approached the significance level.

## 4. Discussion

The simultaneous measurement of plasma CoQ and Chol levels is of interest in assessing the relationship between the presence of these two lipids in the blood and therefore may predict the potential oxidative damage to cholesterol transporter lipoproteins [20,21].

Some genetic and environmental conditions have been associated with a lower level of plasma CoQ values in paediatric patients [10,11,12,13,14,15]. Thus, plasma CoQ status may be a valuable biomarker for certain diseases, both for diagnosis and for the application of therapeutic strategies. Furthermore, the key role of CoQ in protecting Chol transporter lipoproteins against free radical damage strongly advocates the identification of patients with chronic low plasma CoQ values. To our knowledge, previous studies addressing the relationship between dyslipidaemia and CoQ status have rarely been reported in paediatric patients. 

Overall, the CoQ and Chol values were within the reference intervals and balanced in most patients. The explanation of this observation would be that metabolic biosynthetic pathways for CoQ and Chol share common metabolic steps and are tightly regulated [3]. However, eight cases presented low CoQ/Chol ratio values, which suggests dysregulation of CoQ metabolism related to Chol. 

Regarding patients with hypobetalipoproteinemia caused by mutations in the *APOB* gene, the two cases showed low CoQ values, while only one showed a low CoQ/Chol ratio, and the other showed low-normal values (Supplementary Table S1). In this disorder, truncated apoB48 and apoB100 are synthesised, leading to low plasma lipids due to the inability of hepatocytes and enterocytes to assemble normal lipoproteins [22,23]. It has been suggested that an increase in mevalonate pathway activity to increase Chol would lead to a decrease in CoQ biosynthesis in the liver [24]. To our knowledge, no descriptions of the CoQ status in this disease have been reported, but the possibility of CoQ supplementation to correct potential deficits seems advisable. 

Chylomicron retention disease is a rare disease caused by biallelic pathogenic variants in the *SAR1B* gene, leading to a defect in chylomicron secretion by the intestinal tract and ultimately to a defect in lipid absorption and chylomicron retention in the intestine [22,25]. Given that dietary CoQ contributes up to 20% of the total concentration in the blood, this could explain the suboptimal CoQ values, although other mechanisms that explain the unbalanced CoQ/Chol ratio cannot be ruled out, such as increased activity of the mevalonate pathway that can lead to a loss of balance between CoQ and Chol biosynthesis [24]. In any case, current guidelines recommend treatment with liposoluble vitamins, but no results indicating CoQ status have been previously reported in this disease [25].

Hypofibrinogenemia caused by mutations in the *FGG* gene is a rare condition associated with liver disease, caused by the accumulation of mutant fibrinogens within liver cells. Patients display great variability in the severity of liver disease, going from no signs of liver damage to mild/moderate liver fibrosis or cirrhosis [26]. It is usually determined by heterozygous mutations that cause altered secretion of the mutant fibrinogen, which can aggregate within the endoplasmic reticulum of hepatocytes and cause liver damage [26]. Lipid studies have been conducted on hypofibrinogenemia, mainly reporting hypobetalipoproteinemia but providing no data on CoQ status [27]. For this disease, severe liver damage may explain hypocholesterolaemia and the associated low CoQ values [27].

The relationship between total Chol and CoQ values in plasma has been consistently demonstrated [20]. The main conclusion of these studies is that it seems advisable to assess the relationship of CoQ with Chol. Hypercholesterolemia is a pandemic in Western countries, even in paediatric patients. Although there are many studies associating statin treatment with CoQ status, to our knowledge, studies of both CoQ and total Chol in paediatric dyslipidaemia are scarce. Interestingly, our results showed that in most patients the CoQ versus total Chol values were balanced, indicating that supplementation seems unnecessary. Among lipoproteins, the amount of cholesterol in LDL was the most correlated with total CoQ values, supporting the important role of CoQ in preventing LDL oxidation, as previously reported [4,5]. 

Among patients with hypercholesterolemia, five presented low values of the CoQ/Chol ratio (one patient with Alagille syndrome, one with liver cirrhosis, one with heterozygous mutations of the *LDLR* gene, and two of unknown aetiology). Alagille syndrome is a genetic disorder caused by mutations in the jagged canonical Notch ligand 1 (*JAG1*) or in the *NOTCH2* receptor. The disorder causes a deep impact on liver function, in which a reduced number of bile ducts and cholestasis develop shortly after birth, which can lead to cholestasis and liver cirrhosis [28]. Some liver diseases such as cholestasis and cirrhosis present with hypercholesterolemia, explained by a lower metabolic degradation and excretion of cholesterol along with a deficiency of the enzyme lecithin/cholesterol acyltransferase, leading to the formation of an abnormal lipoprotein called LpX that has little or no apolipoprotein B, which accumulates and leads to hypercholesterolemia [29]. The low CoQ values could be explained by this fact because we are comparing CoQ content in a population with normal lipoprotein composition with these cases with aberrant lipoproteins, which probably would contain less CoQ [29]. A similar explanation could be provided for our patient with liver cirrhosis of unknown aetiology. In any case, the results of plasma CoQ status in liver cirrhosis are scarce and controversial, although CoQ supplementation may improve the clinical outcome of cirrhosis and other liver diseases [30,31,32,33,34]. 

The patient with heterozygous *LDLR* mutations showed a very mild reduction in CoQ values. Furthermore, six more patients with mutations in the *LDLR* gene (Supplementary Table S1), showed normal results, suggesting that this was an incidental finding, probably as occurred with the two cases with increased cholesterol of unknown aetiology, where total CoQ values were, in fact, normal and only a very mild imbalance with total Chol could be demonstrated. 

Regarding therapeutic aspects, it has been consistently demonstrated that CoQ supplementation is safe and can prevent free radical damage and improve cardiovascular damage [2,4,5,8]. In the present study, CoQ and Chol values were balanced in most patients. However, the percentage of ubiquinol, the reduced and active antioxidant form of CoQ, is critical to prevent free radical damage. Under CoQ supplementation, as total CoQ increases, so does the ubiquinol value as well, probably explaining this protective effect [4]. Furthermore, a protective effect of CoQ has been demonstrated in liver cirrhosis, suggesting that it could be a coadjuvant therapy for these disorders [30].

The main limitations of this work are related to the size series, especially that of hypobetalipoproteinemia and infantile cirrhosis. Thus, integrating more data seems advisable to increase our knowledge about CoQ and Chol balance in such diseases. Furthermore, we could not analyse the CoQ status in isolated cholesterol transport lipoproteins, so the correlation between CoQ and the different fractions should be carefully assessed. 

## 5. Conclusions

In conclusion, taking our results together and considering the low percentage of impaired CoQ values demonstrated in our series, it seems advisable to analyse plasma CoQ and cholesterol concentrations in patients with hypobetalipoproteinemia and hypercholesterolemia associated with liver damage. Considering that these are chronic diseases and no previous studies have been reported on this issue, analysis of larger series together with CoQ supplementation in these special cases seems advisable.

## Figures and Tables

**Table 1:** Chol, triglycerides, and HDL-LDL and VLDL cholesterol values in patients and controls. In patients, data are reported as median, range, and SD, and in controls as reference intervals (RI).

	Hypercholesterolemia/ Hypertriglyceridemia (n = 54)			Hypobetalipoproteinemia (n = 6)			Reference Values
	µ	Range	SD	µ	Range	SD	RI
CoQ (μmol/L)	1.07	0.34–2.06	0.36	0.2	0.14–0.37	0.097	0.38–1.25
CoQ/Chol (µmol/mol Chol)	171	36.6–362	96	153	81.9–370	109.2	101–280
Chol (mmol/L)	6.63	4.78–22.6	2.48	1.6	1.0–2.2	0.48	2.47–5.2
Triglycerides (mmol/L)	1.57	0.13–28.7	4.05	0.7	0.13–2.22	0.76	0.5–1.85
HDL cholesterol (mmol/L)	1.52	0.76–4.07	0.53	0.7	0.25–1.17	0.36	>1.04
LDL cholesterol (mmol/L)	4.78	2.77–21.2	2.54	0.7	0.52–1.17	0–52	<3.36
VLDL-cholesterol (mmol/L)	0.39	0.12–1.07	0.21	0.3	0.06–1.0	0.34	0.01–0.63

## Data Availability

Data are available as Supplementary Table S1.

## Supplementary Materials

The following supporting information can be downloaded at: https://www.mdpi.com/article/10.3390/antiox13080966/s1. Table S1. Main clinical, biochemical and genetic anonymised data of 60 paediatric patients with dyslipidaemia.
